# Mandelamide Isolated from *Prunus persica* Flowers Attenuates TNF-α–Driven Oxidative and Inflammatory Responses in Human Skin Cells

**DOI:** 10.3390/biom16050672

**Published:** 2026-05-01

**Authors:** Yea Jung Choi, Hee Woon Ann, So-Ri Son, Dae Sik Jang, Sullim Lee

**Affiliations:** 1 College of Korean Medicine, Gachon University, Seongnam 13120, Republic of Korea; domdada22@gachon.ac.kr; 2 Department of Life Science, Gachon University, Seongnam 13120, Republic of Korea; heewoon05@naver.com; 3 College of Pharmacy, Kyung Hee University, 26 Kyungheedae-ro, Dongdaemun-gu, Seoul 02453, Republic of Korea; allosori@khu.ac.kr

**Keywords:** human skin cells, mandelamide, *Prunus persica* flower, reactive oxygen species, skin aging

## Abstract

Skin aging is driven by both intrinsic and extrinsic factors, including ultraviolet (UV) radiation and environmental stressors. Tumor necrosis factor-alpha (TNF-α) is a key pro-aging cytokine that promotes reactive oxygen species (ROS) production, leading to collagen degradation and inflammatory responses in skin cells. In this study, we investigated the protective effects of *Prunus persica* flower extract and its major constituents (**1**–**4**) against TNF-α–induced oxidative and inflammatory responses in human dermal fibroblasts (HDFs) and human epidermal keratinocytes (HEKs). In HDFs, the extract and isolated compounds significantly suppressed TNF-α–induced ROS generation and matrix metalloproteinase-1 (MMP-1) secretion while enhancing collagen synthesis. Notably, mandelamide (**4**) markedly reduced MMP-1 secretion (from 7.53 ± 0.28 to 2.97 ± 0.12, *p* < 0.001) and restored collagen levels (from 3.3 ± 0.03 to 19.1 ± 0.58, *p* < 0.001). In HEKs, mandelamide attenuated the production of inflammatory mediators under TNF-α stimulation and further suppressed MMP expression while restoring the mRNA expression of hyaluronan synthase genes under TNF-α/ interferon-γ (IFN-γ) co-stimulation. Importantly, mandelamide exhibited selective activity under inflammatory conditions without affecting basal cellular states. Collectively, these findings demonstrate that mandelamide is a key bioactive constituent of *Prunus persica* (*P. persica*) flowers and exerts protective effects against inflammation-associated skin aging through the modulation of oxidative stress and extracellular matrix homeostasis.

## 1. Introduction

The skin is the largest organ of the human body and functions as a critical protective barrier against external environmental stressors [1]. However, skin aging is progressively induced by a combination of intrinsic and extrinsic factors, including lifestyle habits, hormonal changes, environmental pollutants, and ultraviolet (UV) radiation [2]. Among these, UV exposure is a major contributor to skin aging through excessive generation of reactive oxygen species (ROS) [3]. Elevated ROS levels disrupt redox homeostasis within skin cells, alter the cutaneous microenvironment, and accelerate cellular and structural aging processes [4,5]. Persistent oxidative stress further promotes the production of pro-inflammatory cytokines, ultimately leading to inflammaging, a chronic low-grade inflammatory state that drives age-associated skin deterioration [6].

Dermal fibroblasts reside primarily in the dermis, whereas keratinocytes are localized in the epidermis. These two cell types communicate through tightly regulated epidermal–dermal crosstalk to maintain skin structure and functional homeostasis [7]. Under inflammatory conditions, however, this intercellular communication is disrupted, resulting in a pro-aging microenvironment that accelerates extracellular matrix (ECM) degradation and impairs skin integrity [8].

Multiple cytokines have been implicated in skin aging, including interleukins (ILs), transforming growth factor-β (TGF-β), interferon-γ (IFN-γ), and tumor necrosis factor-α (TNF-α) [9]. Among these, TNF-α is recognized as a key pro-aging mediator due to its potent ability to induce oxidative stress in both human keratinocytes and human dermal fibroblasts (HDFs) [10]. TNF-α–induced ROS accumulation activates intracellular signaling cascades that upregulate matrix metalloproteinases (MMPs), thereby promoting collagen degradation and ECM remodeling in HDFs [11].

In particular, TNF-α–stimulated ROS activates mitogen-activated protein kinase (MAPK) signaling pathways, leading to increased expression of MMP-1, MMP-2, and MMP-9, as well as pro-inflammatory cytokines such as IL-6, IL-8, and IL-1β [12,13,14]. These molecular events not only contribute to wrinkle formation and skin aging but also exacerbate inflammatory skin disorders, including atopic dermatitis, acne, and psoriasis [15,16,17].

Interferon-γ (IFN-γ) has also been shown to suppress collagen synthesis in human skin fibroblasts by downregulating COL1A1 expression, and co-treatment with TNF-α further potentiates this inhibitory effect, indicating a synergistic suppression of collagen production [18]. In addition, combined TNF-α/IFN-γ stimulation has been widely employed in human keratinocytes to model inflammatory skin conditions and to evaluate the regulation of moisturization-related factors and inflammatory mediators [19,20,21]. Thus, TNF-α/IFN-γ co-stimulation represents a relevant experimental model for assessing inflammatory skin barrier dysfunction and for investigating the skin-protective effects of bioactive compounds.

Excessive ROS generation also contributes to skin moisture loss. Previous studies have demonstrated that UV-induced ROS overproduction suppresses the expression of hyaluronan synthase enzymes (HAS-1, HAS-2, and HAS-3) in skin fibroblasts, leading to reduced hyaluronic acid synthesis and impaired skin hydration and elasticity [22]. Accordingly, inhibition of TNF-α–induced oxidative stress is considered a critical strategy for preventing both structural and functional aspects of skin aging.

Natural compounds with protective effects against TNF-α–induced damage in human dermal fibroblasts have been actively investigated [23,24,25]. Among these, the fruit and flowers of peach (*Prunus persica*) have been reported to exhibit antioxidant and anti-inflammatory properties [26,27]. However, the molecular mechanisms underlying the protective effects of *P. persica* flowers against TNF-α–mediated skin aging remain largely unexplored. Moreover, the biological activities of their major constituents—including prunasin amide, amygdalin amide, prunasin acid, and mandelamide—have not been extensively investigated in human skin cells.

In our previous phytochemical study of *P. persica* flowers, prunasin amide (**1**), amygdalin amide (**2**), prunasin acid (**3**), and mandelamide (**4**) were identified as major polar constituents, along with chlorogenic acid [28]. Notably, mandelic acid–derived cyanogenic glycoside metabolites were particularly abundant in the flower extract, suggesting that these flower-specific metabolites may be closely associated with their biological activity [29].

Therefore, in the present study, we evaluated the protective effects of *P. persica* flower extract and its major constituents (**1–4**) in TNF-α–induced human dermal fibroblasts and further identified mandelamide as the most active compound for subsequent mechanistic investigation. In addition, mandelamide was further evaluated for its inhibitory effects on inflammatory mediators and its skin-protective activity in both TNF-α–induced and TNF-α/IFN-γ–stimulated human epidermal keratinocyte models, thereby demonstrating its dual protective role across distinct human skin cell systems.

## 2. Materials and Methods

### 2.1. Extraction and Isolation

Extraction and isolation of compounds **1**–**4** from *P. persica* flowers were described previously [28]. In brief, the dried *P. persica* flowers (100.0 g) were extracted twice with distilled water at 100 °C for 2 h to obtain the hot water extract (PRPE, 23.5 g). The PRPE was separated via Diaion HP-20 (Mitsubishi, Tokyo, Japan) column chromatography (CC) and eluted with an acetone/H_2_O mixture (40:60 to 60:40 *v*/*v*) to generate eight fractions (F1–F8). Fraction F1 was further separated by Sephadex LH-20 (GE Healthcare, Stockholm, Sweden) CC using a gradient solvent system of H_2_O–acetone, from 100:0 to 60:40 (*v*/*v*), to obtain 10 subfractions (F1-1~F1-10). Subfraction F1-4 was further purified on Sephadex LH-20 (ϕ 3.7 × 49.1 cm) with 40% acetone to afford 13 subfractions (F1-4-1–F1-4-13). Compound **1** (459.8 mg, yield: 1.957%) was isolated from subfraction F1-4-5, compound **2** (19.6 mg, yield: 0.083%) from subfraction F1-4-4, and compounds **3** (99.3 mg, yield: 0.423%) and **4** (125.6 mg, yield: 0.534%) from subfraction F1-4-8 by reversed-phase flash column chromatography on Redi Sep-C18 cartridges (Teledyne Isco., Lincoln, NE, USA).

### 2.2. Cell Culture

Human dermal fibroblasts (HDFs; CAT No. C-12302, adult donor) and human epidermal keratinocytes (HEKs; CAT No. C-12006, pooled adult donor) were purchased from PromoCell GmbH (Heidelberg, Germany). Cells were cultured in Dulbecco’s modified Eagle’s medium (DMEM; CAT No. 10-103-CV, 4.5 g/L glucose) supplemented with 10% fetal bovine serum (FBS) and 1% penicillin–streptomycin under standard culture conditions (37 °C, 5% CO_2_). HDFs and HEKs were used at early passages (P3–P6) for all experiments.

### 2.3. Cell Viability Assay

HDFs were seeded into 96-well plates at a density of 1 × 10^4^ cells/well and incubated for 24 h. Cells were then serum-starved for an additional 24 h and treated with the indicated concentrations of test compounds in serum-free medium for 24 h. Cell viability was assessed using the Ez-Cytox cell viability assay kit (Daeil Lab Service, Seoul, Republic of Korea) according to the manufacturer’s instructions. Briefly, the culture medium was replaced with serum-free DMEM containing 10% Ez-Cytox solution, followed by incubation for 1 h. Absorbance was measured at 450 nm using a SPARK 10M microplate reader (Tecan, Männedorf, Switzerland).

### 2.4. Measurement of Intracellular ROS

HDFs were seeded into black 96-well plates at a density of 1 × 10^4^ cells/well and incubated for 24 h. After serum deprivation for 24 h, cells were pretreated with non-cytotoxic concentrations of the samples for 1 h in serum-free medium and subsequently stimulated with TNF-α (20 ng/mL; PeproTech, Rocky Hill, NJ, USA, CAT No. 300-01A-50UG) in the same serum-free medium in the presence of DCFDA (10 μM) for 15 min. Cells were then washed with Dulbecco’s phosphate-buffered saline (DPBS), and fluorescence intensity was measured at excitation/emission wavelengths of 485/535 nm using a SPARK 10M microplate reader (Tecan, Männedorf, Switzerland).

### 2.5. Enzyme-Linked Immunosorbent Assay (ELISA) in TNF-α-Stimulated HDFs

HDFs were seeded in 48-well plates at a density of 2 × 10^4^ cells/well and incubated for 24 h. The culture medium was replaced with serum-free medium, and cells were incubated for an additional 24 h. Cells were then pretreated with non-cytotoxic concentrations of the samples in serum-free medium for 1 h, followed by stimulation with TNF-α (20 ng/mL) in serum-free medium for 24 h to assess MMP-1 (CAT No. DY901B) and pro-collagen αI (CAT No. DY6220-05). All ELISA kits were purchased from R&D Systems (Minneapolis, MN, USA).

Culture supernatants were collected and analyzed using commercial sandwich ELISA kits according to the manufacturers’ protocols. Absorbance was measured at 450 nm using a SPARK 10M microplate reader.

### 2.6. Enzyme-Linked Immunosorbent Assay (ELISA) in TNF-α-Stimulated HEKs

HEKs were seeded in 48-well plates at a density of 2 × 10^4^ cells/well and incubated for 24 h. The culture medium was then replaced with serum-free medium, and the cells were further incubated for an additional 24 h. Cells were pretreated with the indicated concentrations of test compounds in serum-free medium for 1 h, followed by stimulation with TNF-α (20 ng/mL) in the same serum-free medium for 12 h.

The levels of COX-2, PGE_2_, IL-6, IL-8, and IL-1β in the culture supernatants were measured using commercial sandwich ELISA kits according to the manufacturers’ instructions. The following ELISA kits were used: COX-2 (CAT No. DYC4198-2), PGE_2_ (CAT No. KGE004B), IL-6 (CAT No. DY206), IL-8 (CAT No. DY208), and IL-1β (CAT No. DY401) (R&D Systems, Minneapolis, MN, USA).

After treatment, the culture media were collected and the levels of target proteins were determined using commercial sandwich ELISA kits, following the manufacturer’s instructions. Absorbance at 450 nm was subsequently measured using a SPARK 10M microplate reader.

### 2.7. Radar Plot Analysis

A radar plot was used to compare the biological activities of the extract and isolated compounds based on three parameters: inhibition of MMP-1 secretion, suppression of ROS production, and enhancement of COL1A1 expression. The axes represent the percentage of inhibition (for MMP-1 and ROS) or recovery (for COL1A1) relative to the TNF-α–treated group. For each parameter, activity was normalized to 100% based on the difference between the TNF-α–treated group and the untreated control. Semi-quantitative scores were assigned as follows: 0 (<20%), 1 (20–39%), 2 (40–69%), 3 (70–89%), and 4 (≥90%). Scores from the three parameters were summed to generate a cumulative activity score, which was categorized as follows: 0 (≤3), 1 (4–7), 2 (8–9), and 3 (≥10). Therefore, a larger radar plot area reflects greater overall biological efficacy.

### 2.8. Nitric Oxide (NO) Assay in TNF-α-Stimulated HEKs

HEKs were seeded in 96-well plates at a density of 1 × 10^4^ cells/well and incubated for 24 h. After serum deprivation for 24 h, the cells were pretreated with the indicated concentrations of test compounds in serum-free medium for 1 h, followed by stimulation with TNF-α (20 ng/mL) in the same serum-free medium for 24 h. Culture supernatants were collected, and nitric oxide production was quantified using the Griess assay. Equal volumes of supernatant and Griess reagent (sulfanilamide, N-(1-naphthyl)ethylenediamine dihydrochloride, and phosphoric acid) were mixed and incubated at room temperature for 30 min. Absorbance was measured at 540 nm using a microplate reader (Agilent Technologies, Santa Clara, CA, USA).

### 2.9. Western Blot Analysis

HDFs were seeded in 6-well plates at a density of 3 × 10^5^ cells/well and incubated for 24 h. The cells were then serum-starved overnight, followed by pretreatment with mandelamide at concentrations of 12.5, 25, and 50 μM in serum-free medium for 1 h. Subsequently, the cells were stimulated with TNF-α (20 ng/mL) in the same serum-free medium for 15 min. Cells were washed with DPBS and lysed using RIPA buffer. After centrifugation at 13,000 rpm for 10 min at 4 °C, protein concentrations were determined using a BCA protein assay kit. Equal amounts of protein were separated by SDS–PAGE and transferred to PVDF membranes. Membranes were blocked with 5% skim milk in TBS-T and incubated with primary antibodies overnight at 4 °C, followed by incubation with secondary antibodies for 2 h at room temperature. Protein bands were visualized using the SuperSignal^®^ West Femto chemiluminescence system (Thermo Fisher Scientific, Waltham, MA, USA; Bio-Rad Laboratories, Hercules, CA, USA) and quantified using ImageJ software 1.52a (National Institutes of Health, Bethesda, MD, USA). Phosphorylation levels of ERK, JNK, and p38 were normalized to their respective total protein levels, with GAPDH used as a loading control.

### 2.10. Quantitative Real-Time PCR (qRT-PCR) in TNF-α/IFN-γ–Stimulated HEKs

HEKs were seeded in 6-well plates at a density of 3 × 10^5^ cells/well and incubated for 24 h. After serum deprivation for 24 h, the cells were pretreated with the indicated concentrations of mandelamide in serum-free medium for 1 h, followed by stimulation with TNF-α (20 ng/mL) and IFN-γ (20 ng/mL; PeproTech, Rocky Hill, NJ, USA, Cat. No. 300-02-100UG) in the same serum-free medium for 24 h. Total RNA was extracted using RLT buffer, and RNA concentration was measured using a NanoDrop spectrophotometer (Keen Innovative Solutions, Seoul, Republic of Korea). cDNA was synthesized using a commercial cDNA synthesis kit (Thermo Fisher Scientific, Waltham, MA, USA) according to the manufacturer’s instructions. qRT-PCR was performed using SYBR Green Master Mix on a QuantStudio 3 Real-Time PCR System (Thermo Fisher Scientific, Waltham, MA, USA). Primer sequences are listed in Table 1. Thermal cycling conditions consisted of an initial incubation at 50 °C for 2 min and 95 °C for 10 min, followed by 40 cycles of amplification (95 °C for 15 s and 60 °C for 1 min), and a final dissociation step. The primers used for qRT-PCR were adopted from previously published studies [30,31].

### 2.11. Statistical Analyses

All data are expressed as the mean ± standard error of the mean (SEM). Statistical analyses were performed using one-way analysis of variance (ANOVA) followed by Tukey’s multiple comparisons test in GraphPad Prism version 8.0.1. Differences were considered statistically significant at *p* < 0.05.

## 3. Results

### 3.1. Identification of Compounds ***1***–***4***

**Figure 1:**
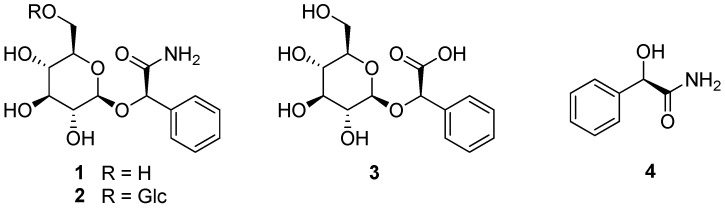
Chemical structures of compounds **1**–**4** isolated from the flowers of *P. persica*.

In our previous study of *P. persica* flowers, compounds **1**–**4** were isolated as major constituents of the PRPE. The isolated compounds were identified as prunasin amide (**1**) [32], amygdalin amide (**2**) [33], prunasin acid (**3**) [32], and mandelamide (**4**) [33] by analysis of spectroscopic data (^1^H and ^13^C NMR) measurements and a comparison with reported data (Figure 1). The corresponding spectra are provided in the Supplementary Materials to support structural identification and assessment of the purity of the isolated compounds (Figures S1–S8).

### 3.2. Effects of the Extract and Compounds ***1***–***4*** on TNF-α–Induced ROS Generation

To exclude cytotoxic effects, non-toxic concentrations of the *P. persica* flower extract and compounds **1**–**4** were first determined in HDFs (Figure S9). These concentrations were subsequently used to evaluate their effects on TNF-α–induced intracellular ROS generation.

**Figure 2:**
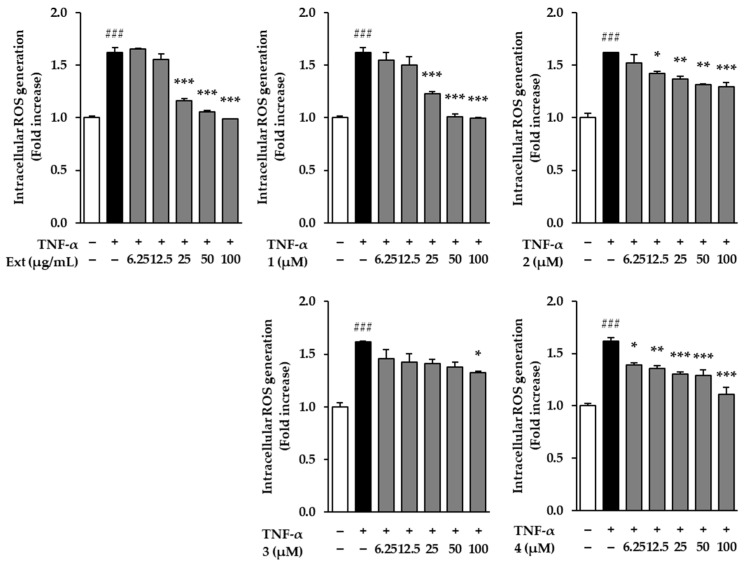
Effects of the *Prunus persica* flower extract and compounds **1–4** on TNF-α–induced intracellular ROS production in human dermal fibroblasts (HDFs). Cells were pretreated with the indicated concentrations of the extract or compounds for 1 h, followed by stimulation with TNF-α (20 ng/mL) in the presence of DCFDA for 15 min. Intracellular ROS levels were quantified by measuring fluorescence intensity at excitation/emission wavelengths of 485/535 nm. Data are expressed as fold changes relative to the untreated control and presented as mean ± SEM (*n* = 3). Statistical significance was determined using one-way ANOVA followed by Tukey’s multiple comparisons test; ### *p* < 0.001 vs. control; *, **, and *** *p* < 0.05, *p* < 0.01, and *p* < 0.001 vs. TNF-α–treated group.

As shown in Figure 2, TNF-α stimulation significantly increased intracellular ROS levels in HDFs (1.62 ± 0.05, *p* < 0.001) compared with the untreated control. Pretreatment with the *P. persica* flower extract markedly attenuated TNF-α–induced ROS generation in a concentration-dependent manner. Specifically, ROS levels were reduced to 1.16 ± 0.02, 1.06 ± 0.01, and 0.99 ± 0.01 at 25, 50, and 100 μg/mL, respectively (all *p* < 0.001).

Among the isolated compounds, prunasin amide (**1**) significantly suppressed ROS production at higher concentrations, decreasing ROS levels to 1.23 ± 0.02, 1.06 ± 0.03, and 1.00 ± 0.01 at 25, 50, and 100 μM, respectively (*p* < 0.001). Amygdalin amide (**2**) also reduced TNF-α–induced ROS generation in a dose-dependent manner, with ROS levels gradually decreasing from 1.52 ± 0.08 at 6.25 μM (*p* < 0.05) to 1.29 ± 0.04 at 100 μM (*p* < 0.001). In contrast, prunasin acid (**3**) exhibited a significant inhibitory effect only at the highest tested concentration, reducing ROS levels to 1.11 ± 0.07 at 100 μM (*p* < 0.05).

Notably, mandelamide (**4**) consistently and significantly inhibited TNF-α–induced ROS generation across all tested concentrations. ROS levels were reduced to 1.39 ± 0.02 (*p* < 0.05), 1.36 ± 0.03 (*p* < 0.01), 1.30 ± 0.02 (*p* < 0.001), 1.29 ± 0.05 (*p* < 0.001), and 1.11 ± 0.07 (*p* < 0.001) at 6.25, 12.5, 25, 50, and 100 μM, respectively. Collectively, these results indicate that mandelamide exhibits the most potent and concentration-independent inhibitory effect on TNF-α–induced oxidative stress among the tested constituents.

### 3.3. Effects of the Extract and Compounds ***1***–***4*** on TNF-α–Induced MMP-1 Secretion and Type I Collagen Production

**Figure 3:**
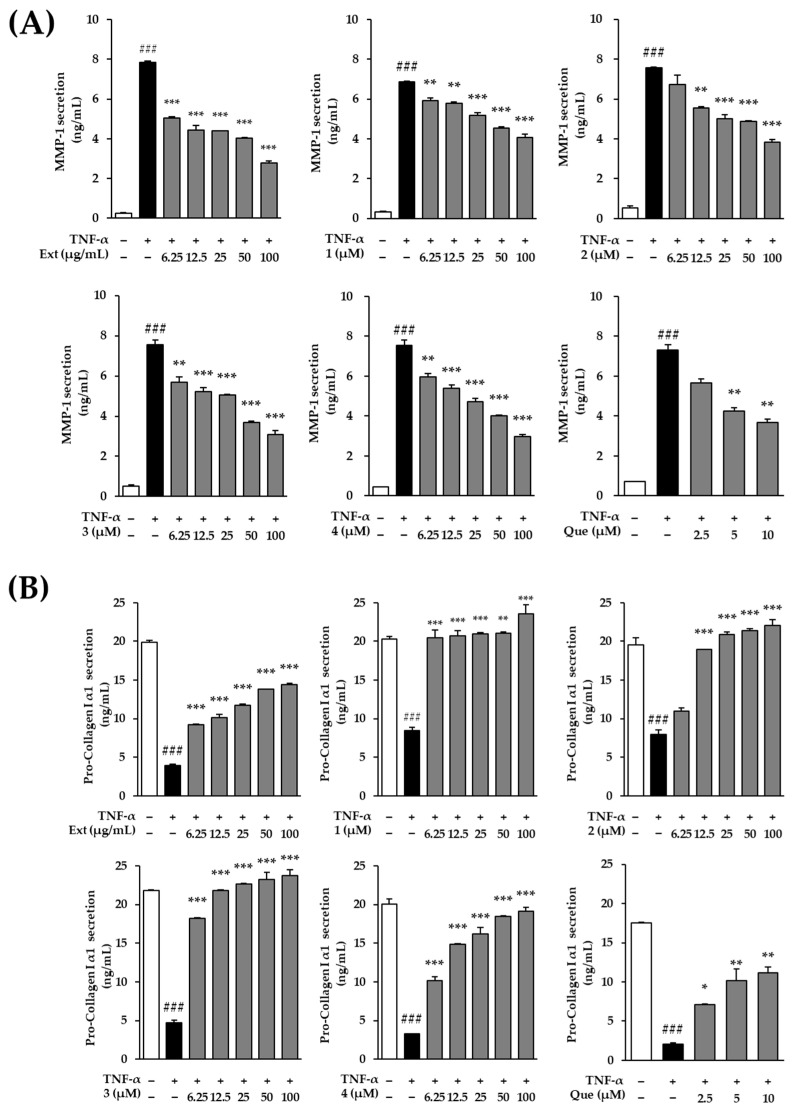
Effects of the *Prunus persica* flower extract and compounds **1**–**4** on TNF-α–induced MMP-1 secretion and type I collagen (COL1A1) production in human dermal fibroblasts (HDFs). Cells were pretreated with the indicated concentrations of the extract or compounds for 1 h, followed by stimulation with TNF-α (20 ng/mL) for 24 h. The secretion levels of (**A**) MMP-1 and (**B**) COL1A1 were quantified using ELISA. Data are expressed as mean ± SEM (*n* = 2). Statistical significance was determined using one-way ANOVA followed by Tukey’s multiple comparisons test; ### *p* < 0.001 vs. control; * *p* < 0.05, ** *p* < 0.01, *** *p* < 0.001 vs. TNF-α–treated group. Quercetin (Que) was used as a positive control.

Next, we evaluated the effects of the *P. persica* flower extract and compounds **1–4** on matrix metalloproteinase-1 (MMP-1) secretion and type I collagen production in TNF-α–stimulated HDFs (Figure 3). In addition, under control conditions (without TNF-α stimulation), neither the extract nor the isolated compounds, including mandelamide, induced significant changes in MMP-1 secretion (Figure S10).

TNF-α stimulation markedly increased MMP-1 secretion compared with the untreated control (7.83 ± 0.08, *p* < 0.001). Pretreatment with the *P. persica* flower extract significantly suppressed TNF-α–induced MMP-1 secretion across all tested concentrations, reducing MMP-1 levels to 5.05 ± 0.07, 4.45 ± 0.23, 4.42 ± 0.00, 4.03 ± 0.02, and 2.77 ± 0.12 at 6.25, 12.5, 25, 50, and 100 μg/mL, respectively (all *p* < 0.001).

Among the isolated compounds, prunasin amide (**1**) and amygdalin amide (**2**) significantly attenuated MMP-1 secretion in a concentration-dependent manner. Prunasin amide (**1**) reduced MMP-1 levels to 5.92 ± 0.13 (*p* < 0.01), 5.78 ± 0.07 (*p* < 0.01), 5.17 ± 0.15 (*p* < 0.001), 4.53 ± 0.08 (*p* < 0.001), and 4.07 ± 0.18 (*p* < 0.001) at increasing concentrations, compared with TNF-α stimulation (6.85 ± 0.03, *p* < 0.001). Similarly, amygdalin amide (**2**) decreased MMP-1 secretion to 5.55 ± 0.07 (*p* < 0.01), 5.00 ± 0.22 (*p* < 0.001), 4.88 ± 0.03 (*p* < 0.001), and 3.83 ± 0.12 (*p* < 0.001) at concentrations ranging from 12.5 to 100 μM, compared with TNF-α stimulation (7.57 ± 0.03, *p* < 0.001).

Prunasin acid (**3**) also exhibited a strong inhibitory effect on TNF-α–induced MMP-1 secretion across all tested concentrations, reducing MMP-1 levels to 5.70 ± 0.25 (*p* < 0.01), 5.22 ± 0.20 (*p* < 0.01), 5.07 ± 0.02 (*p* < 0.001), 3.70 ± 0.05 (*p* < 0.001), and 3.08 ± 0.20 (*p* < 0.001), compared with TNF-α stimulation (7.57 ± 0.22, *p* < 0.001). Notably, mandelamide (**4**) consistently and robustly suppressed MMP-1 secretion at all tested concentrations, with MMP-1 levels decreasing to 5.95 ± 0.20, 5.40 ± 0.15, 4.73 ± 0.15, 4.00 ± 0.05, and 2.97 ± 0.12 at 6.25–100 μM, respectively, compared with TNF-α stimulation (7.57 ± 0.28, *p* < 0.001) (all *p* < 0.001).

In parallel, TNF-α stimulation markedly reduced type I collagen production (3.95 ± 0.13, *p* < 0.001). Pretreatment with *P. persica* flower extract significantly restored collagen levels in a concentration-dependent manner, increasing collagen production to 9.22 ± 0.05, 10.17 ± 0.40, 11.77 ± 0.15, 13.85 ± 0.02, and 14.40 ± 0.22 at 6.25–100 μg/mL, respectively (all *p* < 0.001).

Consistent with their effects on MMP-1, the isolated compounds differentially enhanced collagen production. Prunasin amide (**1**) moderately increased collagen levels to 20.42 ± 1.05, 20.75 ± 0.68, 20.97 ± 0.15, 21.02 ± 0.15, and 23.60 ± 1.18 across increasing concentrations compared with TNF-α stimulation (8.47 ± 0.40, *p* < 0.001) (all *p* < 0.001). Amygdalin amide (**2**) similarly elevated collagen production to 11.00 ± 0.38, 18.95 ± 0.02, 20.90 ± 0.28, 21.40 ± 0.23, and 22.02 ± 0.75 compared with TNF-α stimulation (7.97 ± 0.60, *p* < 0.001) (all p < 0.001). Prunasin acid (**3**) exhibited a pronounced stimulatory effect, restoring collagen levels to near-control values at higher concentrations (18.25 ± 0.03 to 23.75 ± 0.78, *p* < 0.001), compared with TNF-α stimulation (4.75 ± 0.33, *p* < 0.001).

Notably, mandelamide (**4**) showed the most robust enhancement of collagen production among the tested compounds, increasing collagen levels to 10.17 ± 0.50, 14.85 ± 0.13, 16.20 ± 0.83, 18.45 ± 0.13, and 19.10 ± 0.58 at 6.25–100 μM, respectively, compared with TNF-α stimulation (3.30 ± 0.03, *p* < 0.001) (all *p* < 0.001). Collectively, these results indicate that mandelamide most effectively suppresses TNF-α–induced collagen degradation while promoting collagen synthesis, supporting its selection for subsequent mechanistic analyses.

### 3.4. Radar Plot Analysis for Comparative Evaluation of the Extract and Compounds ***1***–***4*** in TNF-α–Induced HDFs

**Figure 4:**
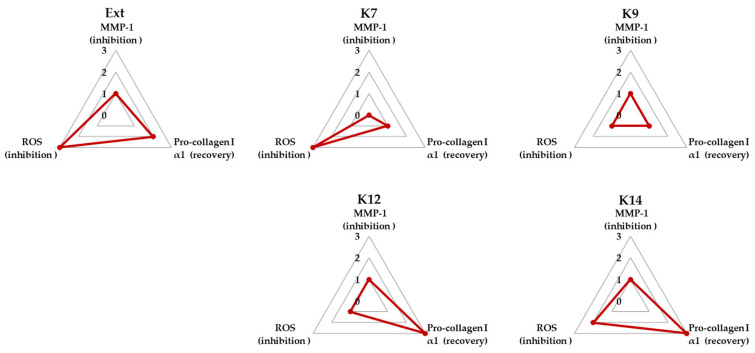
Radar plot analysis comparing the biological activities of *Prunus persica* flower extract and compounds **1**–**4** in TNF-α–stimulated human dermal fibroblasts (HDFs). The analysis was based on the inhibition of ROS production and MMP-1 secretion and the enhancement of COL1A1 expression. The axes represent the percentage of inhibition (for ROS and MMP-1) or recovery (for COL1A1) relative to the TNF-α–treated group. Activity was normalized based on the difference between the TNF-α–treated and untreated control groups. Semi-quantitative scores (0–4) were assigned based on the magnitude of biological response: 0 (<20%, minimal effect), 1 (20–39%, weak effect), 2 (40–69%, moderate effect), 3 (70–89%, strong effect), and 4 (≥90%, near-complete effect). A larger radar plot area indicates greater overall efficacy.

To facilitate an integrated comparison of the biological activities of the *P. persica* flower extract and compounds **1**–**4**, a radar plot analysis was performed using three key readouts relevant to TNF-α–induced fibroblast damage: suppression of intracellular ROS generation, inhibition of MMP-1 secretion, and enhancement of pro-collagen type I alpha 1 expression (Figure 4).

As illustrated in Figure 4, the extract and prunasin amide (**1**) exhibited relatively strong inhibitory effects on ROS generation. In contrast, the inhibitory effects on MMP-1 secretion were largely comparable among the extract and all four compounds, with no pronounced differences observed across the tested samples. Notably, the extract as well as compounds **3** (prunasin acid) and **4** (mandelamide) showed the most prominent enhancement of pro-collagen type I alpha 1 expression.

When the three parameters were considered collectively, mandelamide (**4**) demonstrated the most balanced and robust overall activity, characterized by concurrent suppression of oxidative stress and MMP-1 secretion together with promotion of pro-collagen type I alpha 1 production. Based on this integrated evaluation, mandelamide was selected for subsequent mechanistic analyses.

### 3.5. The Effects of Mandelamide on TNF-α-Induced MAPK Phosphorylation

**Figure 5:**
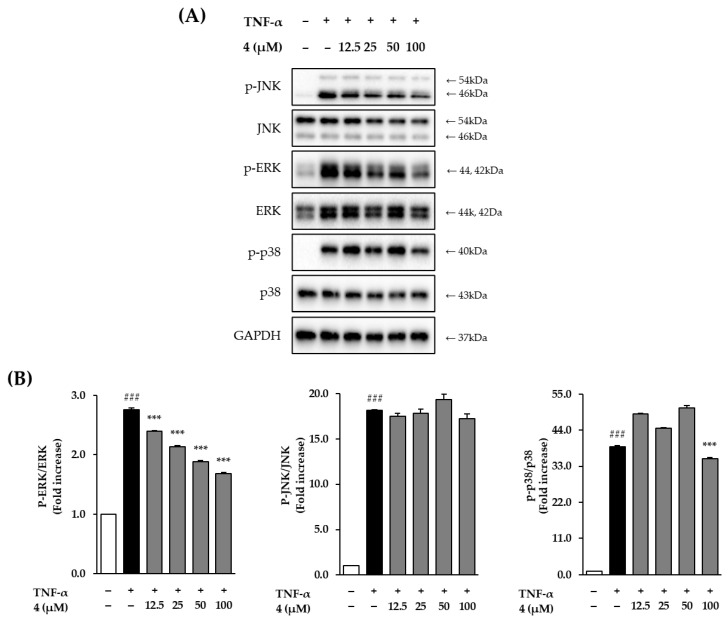
Effects of mandelamide (**4**) on MAPK phosphorylation in TNF-α–stimulated human dermal fibroblasts (HDFs). (**A**) Cells were pretreated with mandelamide at the indicated concentrations (12.5, 25, 50, and 100 μM) for 1 h, followed by stimulation with TNF-α for 15 min. Phosphorylation and total protein levels of ERK, JNK, and p38 were analyzed by Western blotting, with GAPDH used as a loading control. (**B**) Quantitative data are expressed as fold changes relative to the untreated control and presented as mean ± SEM (*n* = 3). Statistical significance was determined using one-way ANOVA followed by Tukey’s multiple comparisons test; ### *p* < 0.001 vs. control; *** *p* < 0.001 vs. TNF-α–treated group. Original western blots can be found at Supplementary Materials.

Based on its superior antioxidant and anti-inflammatory effects, mandelamide (**4**) was further evaluated for its effects on mitogen-activated protein kinase (MAPK) signaling in TNF-α–stimulated HDFs (Figure 5).

TNF-α stimulation significantly increased the phosphorylation of MAPKs, with the most pronounced activation observed in the p38 pathway, followed by JNK, while ERK showed the lowest relative level of induction compared with the untreated control.

Despite its relatively lower induction, ERK phosphorylation was compared with the untreated control (2.76 ± 0.03, *p* < 0.001). Pretreatment with mandelamide significantly and concentration-dependently suppressed ERK phosphorylation, reducing phosphorylation levels to 2.39 ± 0.01, 2.13 ± 0.02, 1.88 ± 0.03, and 1.68 ± 0.02 at 12.5, 25, 50, and 100 μM, respectively (all *p* < 0.001).

In contrast, mandelamide did not significantly affect TNF-α–induced JNK phosphorylation, which remained elevated compared with the control (18.14 ± 0.12, *p* < 0.001). With respect to p38 signaling, mandelamide exerted a modest inhibitory effect only at the highest tested concentration, reducing p38 phosphorylation from 38.94 ± 0.37 to 35.35 ± 0.37 at 100 μM (*p* < 0.001).

Collectively, these results indicate that mandelamide selectively attenuates TNF-α–induced MAPK activation, preferentially targeting ERK despite its relatively lower induction, with comparatively limited effects on JNK and p38 signaling.

### 3.6. Effects of Mandelamide on Pro-Inflammatory Mediator Secretion in TNF-α-Stimulated Human Epidermal Keratinocytes

**Figure 6:**
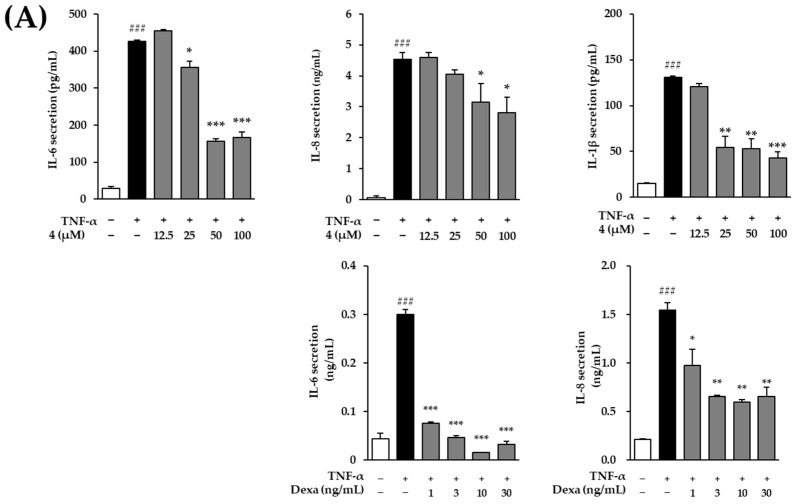

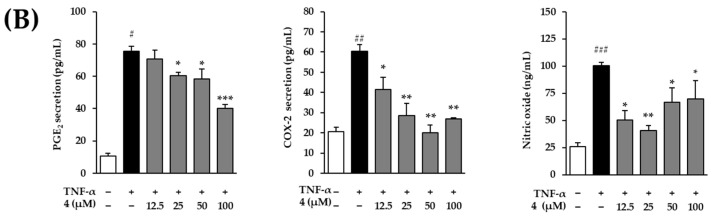
Effects of mandelamide (**4**) on pro-inflammatory mediator secretion in TNF-α–stimulated human epidermal keratinocytes (HEKs). Cells were pretreated with mandelamide at the indicated concentrations (12.5, 25, 50, and 100 μM) for 1 h, followed by stimulation with TNF-α for 12 or 24 h. The secretion levels of IL-6, IL-8, and IL-1β (**A**), as well as COX-2, PGE_2_, and nitric oxide (NO) (**B**), were quantified using ELISA, while NO production was additionally measured using the Griess assay. All experiments were performed according to the manufacturers’ protocols. Data are presented as mean ± SEM (*n* = 3). Statistical significance was determined using one-way ANOVA followed by Tukey’s multiple comparisons test; # *p* < 0.05, ## *p* < 0.01, and ### *p* < 0.001 vs. control; * *p* < 0.05, ** *p* < 0.01, and *** *p* < 0.001 vs. TNF-α–treated group. Dexamethasone (Dexa) was used as a positive control in the IL-6 and IL-8 ELISAs.

To evaluate the anti-inflammatory effects of mandelamide (**4**) in an inflammatory keratinocyte model, its effects on pro-inflammatory cytokine secretion were examined in TNF-α–stimulated human epidermal keratinocytes (HEKs) using ELISA and Griess assays (Figure 6).

As shown in Figure 6A, TNF-α stimulation markedly increased IL-6 secretion compared with the untreated control (426.5 ± 2.55 pg/mL, *p* < 0.001). Pretreatment with mandelamide significantly reduced IL-6 levels in a concentration-dependent manner, decreasing secretion to 355.5 ± 16.45 pg/mL (*p* < 0.05), 156.0 ± 7.14 pg/mL (*p* < 0.001), and 165.8 ± 16.16 pg/mL (*p* < 0.001) at 25, 50, and 100 μM, respectively. IL-8 secretion was also significantly suppressed at 50 and 100 μM, with levels reduced to 3.14 ± 0.62 and 2.81 ± 0.49 ng/mL, respectively (*p* < 0.05), compared with the TNF-α-treated group (4.54 ± 0.22 ng/mL, *p* < 0.001). Similarly, IL-1β secretion was markedly decreased by mandelamide at 25, 50, and 100 μM, reducing levels from 130.6 ± 1.54 pg/mL (*p* < 0.001) to 54.5 ± 11.98, 53.1 ± 10.47, and 43.1 ± 6.32 pg/mL, respectively (*p* < 0.01 or *p* < 0.001).

Consistent with these effects, mandelamide also attenuated downstream inflammatory mediators associated with cyclooxygenase signaling (Figure 6B). PGE_2_ secretion induced by TNF-α (75.5 ± 3.14 pg/mL, *p* < 0.05) was significantly reduced to 60.5 ± 2.08, 58.4 ± 6.15, and 40.3 ± 2.15 pg/mL at 25, 50, and 100 μM, respectively (*p* < 0.05 or *p* < 0.001). In parallel, COX-2 secretion was significantly decreased by mandelamide at 12.5, 25, 50, and 100 μM, with levels reduced to 41.4 ± 6.18 (*p* < 0.05), 28.5 ± 6.01 (*p* < 0.01), 20.2 ± 3.59 (*p* < 0.01), and 26.9 ± 0.54 pg/mL (*p* < 0.01), respectively, compared with the TNF-α-treated group (60.5 ± 3.14 pg/mL, *p* < 0.01).

Moreover, mandelamide significantly suppressed nitric oxide (NO) production induced by TNF-α stimulation. NO levels were reduced from 100.6 ± 3.04 pg/mL (*p* < 0.001) to 50.1 ± 9.12 (*p* < 0.05), 40.7 ± 4.50 (*p* < 0.01), 66.6 ± 13.46 (*p* < 0.05), and 70.0 ± 16.65 pg/mL (*p* < 0.05) at 12.5, 25, 50, and 100 μM, respectively.

Collectively, these results demonstrate that mandelamide effectively suppresses TNF-α-induced inflammatory mediator production in human epidermal keratinocytes, supporting its anti-inflammatory activity in epidermal inflammatory conditions.

### 3.7. Effects of Mandelamide on the mRNA Expression of MMPs and Collagen-Related Genes in TNF-α/IFN-γ–Stimulated Human Epidermal Keratinocytes

**Figure 7:**
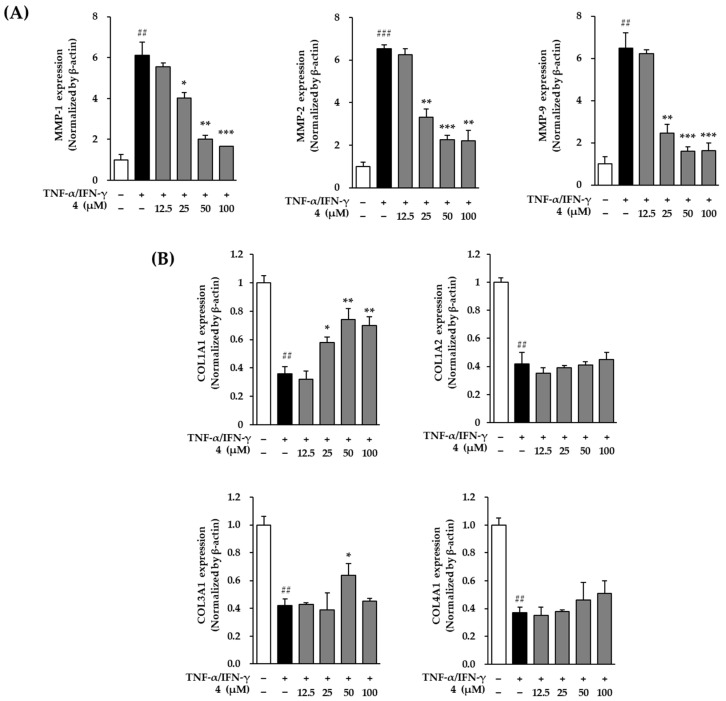
Effects of mandelamide (**4**) on the mRNA expression of MMPs and collagen-related genes in TNF-α/IFN-γ–stimulated human epidermal keratinocytes (HEKs). Cells were pretreated with mandelamide at the indicated concentrations (12.5, 25, 50, and 100 μM) for 1 h, followed by stimulation with TNF-α/IFN-γ for 24 h. The mRNA expression levels of MMP-1, MMP-2, MMP-9 (**A**), as well as COL1A1, COL1A2, COL3A1, and COL4A1 (**B**) were quantified by quantitative real-time PCR (qRT-PCR). All experiments were performed according to the manufacturers’ protocols. Data are presented as mean ± SEM (*n* = 3). Statistical significance was determined using one-way ANOVA followed by Tukey’s multiple comparisons test; ## *p* < 0.05 and ### *p* < 0.001 vs. control; *, **, and *** *p* < 0.05, *p* < 0.01, and *p* < 0.001 vs. TNF-α/IFN-γ–treated group.

To further examine the regulatory effects of mandelamide (**4**) on wrinkle- and matrix remodeling–related gene expression, the mRNA levels of matrix metalloproteinases (MMPs) and collagen-related genes were analyzed in TNF-α/IFN-γ–stimulated human epidermal keratinocytes (HEKs) using quantitative real-time PCR (Figure 7).

As shown in Figure 7A, TNF-α/IFN-γ stimulation markedly upregulated the expression of wrinkle-associated MMP genes. Pretreatment with mandelamide significantly suppressed MMP-1 mRNA expression in a concentration-dependent manner, reducing expression levels from 6.11 ± 0.66 (*p* < 0.01) to 4.02 ± 0.26 (*p* < 0.05), 2.02 ± 0.18 (*p* < 0.01), and 1.65 ± 0.40 (*p* < 0.001) at 25, 50, and 100 μM, respectively. Similarly, MMP-2 expression was significantly reduced from 6.54 ± 0.16 (*p* < 0.001) to 3.32 ± 0.37 (*p* < 0.01), 2.25 ± 0.21 (*p* < 0.001), and 2.19 ± 0.51 (*p* < 0.01), while MMP-9 expression decreased from 6.48 ± 0.72 (*p* < 0.01) to 2.47 ± 0.40 (*p* < 0.01), 1.59 ± 0.22 (*p* < 0.001), and 1.64 ± 0.34 (*p* < 0.001) at the corresponding concentrations.

In parallel, mandelamide restored the expression of collagen-related genes that were suppressed by TNF-α/IFN-γ stimulation (Figure 7B). COL1A1 mRNA expression was significantly increased from 0.36 ± 0.05 (*p* < 0.01) to 0.58 ± 0.04 (*p* < 0.05), 0.74 ± 0.08 (*p* < 0.01), and 0.70 ± 0.06 (*p* < 0.01) at 25, 50, and 100 μM, respectively. COL3A1 expression was also significantly elevated at 50 μM (0.64 ± 0.08, *p* < 0.01). In contrast, the expression levels of COL1A2 and COL4A1 were not significantly altered by mandelamide treatment.

Collectively, these results indicate that mandelamide suppresses TNF-α/IFN-γ–induced expression of matrix-degrading enzymes while partially restoring collagen-related gene expression in keratinocytes.

### 3.8. Effects of Mandelamide on the mRNA Expression of Hyaluronan Synthase Genes in TNF-α/IFN-γ–Stimulated Human Epidermal Keratinocytes

**Figure 8:**
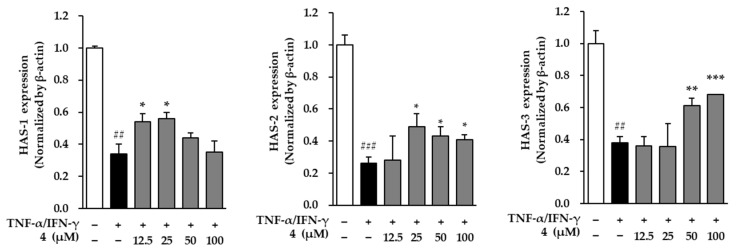
Effects of mandelamide (**4**) on hyaluronan synthase (HAS) gene expression in TNF-α/IFN-γ–stimulated human epidermal keratinocytes (HEKs). Cells were pretreated with mandelamide at the indicated concentrations (12.5, 25, 50, and 100 μM) for 1 h, followed by stimulation with TNF-α/IFN-γ for 24 h. The mRNA expression levels of HAS-1, HAS-2, and HAS-3 were quantified by quantitative real-time PCR (qRT-PCR). All experiments were performed according to the manufacturers’ protocols. Data are presented as mean ± SEM (*n* = 3). Statistical significance was determined using one-way ANOVA followed by Tukey’s multiple comparisons test; ## *p* < 0.05 and ### *p* < 0.001 vs. control; *, **, and *** *p* < 0.05, *p* < 0.01, and *p* < 0.001 vs. TNF-α/IFN-γ–treated group.

To further investigate the effects of mandelamide (**4**) on skin hydration–related gene expression, the mRNA levels of hyaluronan synthase (HAS) isoforms were analyzed in TNF-α/IFN-γ–stimulated human epidermal keratinocytes (HEKs) using quantitative real-time PCR (Figure 8).

As shown in Figure 8, TNF-α/IFN-γ stimulation significantly suppressed the expression of HAS genes, whereas mandelamide treatment markedly restored their expression in a concentration-dependent manner. HAS-1 mRNA expression was increased from 0.34 ± 0.06 (*p* < 0.01) to 0.54 ± 0.05 (*p* < 0.05) and 0.56 ± 0.04 (*p* < 0.01) at 12.5 and 25 μM, respectively. HAS-2 expression was significantly elevated to 0.49 ± 0.08, 0.43 ± 0.06, and 0.41 ± 0.03 at 25, 50, and 100 μM, respectively (all *p* < 0.05). In addition, HAS-3 mRNA expression was significantly increased to 0.61 ± 0.05 (*p* < 0.01) and 0.68 ± 0.04 (*p* < 0.001) at 50 and 100 μM, respectively.

Collectively, these results indicate that mandelamide effectively restores the expression of hyaluronan synthase genes suppressed under inflammatory conditions, suggesting its potential role in improving epidermal hydration-related capacity.

## 4. Discussion

The skin is a multilayered organ composed of the epidermis, dermis, and adipose tissue, and it undergoes progressive aging in response to both intrinsic and extrinsic stressors [34,35]. Among various inflammatory mediators, tumor necrosis factor-α (TNF-α) is a well-established inducer of oxidative stress in skin cells [36]. Sustained TNF-α stimulation elevates intracellular reactive oxygen species (ROS) levels, thereby promoting skin aging through the induction of matrix metalloproteinases (MMPs), including MMP-1 and MMP-3, which are responsible for collagen degradation in dermal fibroblasts [37]. In addition to TNF-α alone, co-stimulation with other pro-inflammatory cytokines such as interferon-γ (IFN-γ), IL-22, and IL-17A has been shown to amplify inflammatory signaling and accelerate skin dysfunction [38,39,40]. In particular, IFN-γ has been widely employed to establish in vitro models that recapitulate chronic or atopic inflammatory skin microenvironments [41,42,43]. Thus, evaluating skin-protective effects under diverse cytokine-driven inflammatory conditions is essential for understanding their relevance to complex inflammatory skin aging.

Oxidative stress plays a central role in the initiation and progression of skin aging, making the suppression of ROS a critical therapeutic target. In the present study, *P. persica* flower extract and its isolated compounds significantly attenuated TNF-α–induced ROS generation in human dermal fibroblasts. Notably, mandelamide (**4**) consistently exhibited strong inhibitory effects across all tested concentrations, indicating a robust antioxidant capacity under inflammatory conditions (Figure 2).

Among MMP family members, MMP-1 serves as the primary enzyme responsible for initiating the degradation of type I collagen, the most abundant structural protein in the dermal extracellular matrix [44]. Following initial cleavage by MMP-1, other MMPs, including MMP-2 and MMP-9, further degrade collagen fragments and additional extracellular matrix components such as gelatin and elastin [45]. This sequential degradation contributes to dermal thinning, loss of elasticity, and wrinkle formation during skin aging [46]. In this study, both the extract and isolated compounds suppressed TNF-α–induced MMP-1 secretion while simultaneously restoring type I collagen production (Figure 3). Consistent with the integrated radar plot analysis (Figure 4), mandelamide was selected as the lead candidate, as it exhibited consistently stronger inhibitory effects on intracellular ROS levels and MMP-1 secretion across the tested concentration range compared with the other compounds, along with a concurrent restoration of pro-collagen type I α1 production.

At the transcriptional level, mandelamide significantly downregulated the mRNA expression of MMP-1, MMP-2, and MMP-9 in TNF-α/IFN-γ–stimulated human epidermal keratinocytes, further supporting its inhibitory effects on matrix degradation pathways (Figure 7). Collagen homeostasis is regulated by multiple collagen subtypes, among which collagen type I (COL1A1 and COL1A2) is essential for dermal strength and elasticity, while collagen type III (COL3A1) plays a key role during tissue repair and remodeling [47,48]. Although collagen type IV (COL4A1) is not a major dermal collagen, it contributes to basement membrane integrity and epidermal–dermal interactions [49]. Excessive ROS generation during skin aging disrupts the expression of these collagens, leading to compromised barrier function and increased vulnerability to environmental stressors [50]. In line with this, mandelamide restored the expression of COL1A1 and COL3A1 under inflammatory conditions, while no significant changes were observed in COL1A2 and COL4A1. These findings suggest that the restorative effects of mandelamide are primarily associated with fibrillar collagens rather than basement membrane components.

MAPK signaling pathways act as critical upstream regulators of collagen degradation and inflammatory responses in dermal fibroblasts. TNF-α–induced activation of MAPKs promotes MMP expression and accelerates extracellular matrix breakdown [51,52]. Consistent with our results, TNF-α–induced MAPK activation was predominantly mediated by the p38 pathway, followed by JNK, while ERK showed a relatively lower level of induction.

Despite this hierarchy, mandelamide selectively attenuated TNF-α–induced ERK phosphorylation, with comparatively limited effects on JNK and p38 signaling (Figure 5). This selective modulation suggests that mandelamide does not broadly suppress MAPK signaling but may preferentially target specific pro-aging pathways associated with ERK activation.

ROS-mediated activation of transcription factors such as activator protein-1 (AP-1), composed of FOS and c-JUN, further amplifies inflammatory signaling cascades [53]. Activated MAPKs and AP-1 can subsequently engage downstream pathways, including NF-κB, leading to the upregulation of pro-inflammatory mediators such as COX-2, PGE_2_, nitric oxide (NO), IL-6, IL-8, and IL-1β [30,54]. In agreement with this mechanism, mandelamide significantly reduced the secretion of these inflammatory mediators in TNF-α–stimulated keratinocytes (Figure 6), supporting its anti-inflammatory activity at both upstream signaling and downstream effector levels.

Beyond its anti-wrinkle and anti-inflammatory effects, mandelamide also significantly upregulated the mRNA expression of hyaluronan synthase isoforms HAS1, HAS2, and HAS3 (Figure 8). Hyaluronic acid synthesized by these enzymes plays a crucial role in maintaining epidermal hydration and skin elasticity. Thus, the observed restoration of HAS expression suggests that mandelamide may contribute to improving skin hydration capacity in addition to mitigating inflammatory skin aging.

Although the biological effects of mandelamide itself have not been extensively characterized in skin models, related species within the *Prunus* genus provide supporting evidence. Previous studies have shown that *Prunus mume* extract increases SIRT1 expression and suppresses MMP-1 in UV-induced skin aging models [55], as well as enhances hyaluronic acid production in UVB-stimulated human keratinocytes [56]. These findings support the notion that *P. persica* flower and its constituents may exert comparable protective effects against skin aging.

Despite these findings, several limitations of the present study should be acknowledged. First, although both dermal fibroblasts and epidermal keratinocytes were examined, the skin is a highly integrated organ in which dynamic crosstalk between multiple cell types plays a critical role in aging and inflammation [57]. Future studies employing three-dimensional reconstructed human skin models that incorporate both fibroblasts and keratinocytes may provide a more comprehensive evaluation. Indeed, previous studies have successfully applied such models under cytokine-induced inflammatory conditions, including IL-4/IL-13 and TNF-α/IFN-γ stimulation [58].

Second, the current study was conducted exclusively under in vitro conditions. Additional in vivo studies are required to validate the protective effects of mandelamide in physiological contexts. Animal models of skin aging, combined with histological analyses such as hematoxylin and eosin staining, would allow for direct assessment of tissue-level changes and signaling mechanisms, including MAPK activation, following topical or systemic application of mandelamide [59,60].

Third, the prolonged serum-free conditions used in this study may represent an additional limitation. Although no significant cytotoxicity was observed, extended serum starvation may have influenced the basal cellular state and responsiveness to inflammatory stimuli. Furthermore, it should be noted that the WST-1 (Ez-Cytox) assay reflects mitochondrial dehydrogenase activity, which can be influenced by metabolic shifts under starvation rather than strictly representing cell number or biomass. Since ROS levels (Figure 2) were not normalized to total protein content (e.g., via BCA assay), the observed reduction may have been partially affected by minor variations in cellular biomass or altered metabolic rates. Consequently, these findings should be interpreted with caution, acknowledging the potential impact of the cellular metabolic state under prolonged starvation conditions.

## 5. Conclusions

In conclusion, this study investigated the protective effects of *Prunus persica* flower extract and its isolated compounds against TNF-α–induced oxidative stress and inflammatory responses in human skin cells. The results demonstrate that *P. persica* flower extract and its constituents attenuate TNF-α–induced oxidative stress and inflammatory responses in skin cell models.

Among the tested constituents, mandelamide (**4**) consistently exhibited the most pronounced effects. Mandelamide suppressed intracellular ROS production, reduced the expression and secretion of matrix metalloproteinases (MMP-1, MMP-2, and MMP-9), and selectively inhibited ERK phosphorylation, with comparatively limited effects on p38 signaling, thereby contributing to the preservation of collagen homeostasis.

In addition, mandelamide decreased the production of pro-inflammatory mediators and restored the expression of hyaluronan synthase enzymes (HAS1, HAS2, and HAS3), indicating a potential role in maintaining epidermal hydration-related functions under inflammatory conditions.

Although further validation in more complex skin models and in vivo systems is required, these findings provide experimental evidence supporting the skin-protective effects of *P. persica* flower–derived mandelamide against inflammation-associated skin aging.

## Figures and Tables

**Table 1:** Primer sequences.

Gene	Sense Primer Sequence (5′-3′)	Antisense Primer Sequence (5′-3′)
*MMP-1* (*AF158733*)	ATTCTACTGATATCGGGGCTTT	ATGTCCTTGGGGTATCCGTGTA
*MMP-2* (*NM_004530*)	CAGGGAATGAGTACTGGGTCTATT	ACTCCAGTTAAAGGCAGCATCTAC
*MMP-9* (*NM_004994*)	CACTGTCCACCCCTCAGAGC	CACTTGTCGGCGATAAGG
*COL1A1* (*X07884*)	CTCGAGGTGGACACCACCCT	CAGCTGGATGGCCACATCGG
*COL1A2* (*NM_000089*)	AGAAACACGTCTGGCTAGGAG	GCATGAAGGCAAGTTGGGTAG
*COL3A1* (*NM_000090*)	GTTTTGCCCCGTATTATGGA	GGAAGTTCAGGATTGCCGTA
*COL4A1* (*NM_001845*)	ACTCTTTTGTGATGCACACCA	AAGCTGTAAGCGTTTGCGTA
*HAS-1* (*NM_001523*)	CCACCCAGTACAGCGTCAAC	CATGGTGCTTCTGTCGCTC
*HAS-2* (*NM_005328*)	TTTGTTCAAGTCCCAGCAGC	ATCCTCCTGGGTGGTGTGAT
*HAS-3* (*NM_005329*)	CCCAGCCAGATTTGTTGATG	AGTGGTCACGGGTTTCTTCC
*β-actin* (*DQ407611*)	AGAGATGGCCACGGCTGCTT	ATTTGCGGTGGACGATGGAG

## Data Availability

All data generated or analyzed during this study are included in this published article.

## Supplementary Materials

The following supporting information can be downloaded at: https://www.mdpi.com/article/10.3390/biom16050672/s1, Figure S1: ^1^H-NMR spectrum of compound **1** (500 MHz, MeOD-*d*_4_), Figure S2: ^13^C-NMR spectrum of compound **1** (125 MHz, MeOD-*d*_4_), Figure S3: ^1^H-NMR spectrum of compound **2** (500 MHz, D_2_O), Figure S4: ^13^C-NMR spectrum of compound **2** (125 MHz, D_2_O), Figure S5: ^1^H-NMR spectrum of compound **3** (500 MHz, MeOD-*d*_4_), Figure S6: ^13^C-NMR spectrum of compound **3** (125 MHz, MeOD-*d*_4_), Figure S7: ^1^H-NMR spectrum of compound **4** (500 MHz, D_2_O), Figure S8. ^13^C-NMR spectrum of compound **4** (125 MHz, D_2_O), Figure S9: Effects of the *Prunus persica* flower extract and compounds **1**–**4** on cell viability in human dermal fibroblasts (HDFs), Figure S10: Effects of the *Prunus persica* flower extract and compounds **1**–**4** on MMP-1 secretion in human dermal fibroblasts (HDFs). File S1: Original western blots.

## Abbreviations

AP-1	Activator protein 1
COL1A1	Collagen type I alpha 1 chain
COL1A2	Collagen type I alpha 2 chain
COL3A1	Collagen type III alpha 1 chain
COL4A1	Collagen type IV alpha 1 chain
COX-2	Cyclooxygenase-2
c-Jun	Jun proto-oncogene
DCFDA	2′,7′-Dichlorodihydrofluorescein diacetate
DMEM	Dulbecco’s modified Eagle’s medium
DPBS	Dulbecco’s phosphate-buffered saline
ECM	Extracellular matrix
ELISA	Enzyme-linked immunosorbent assay
ERK	Extracellular signal-regulated kinase
FBS	Fetal bovine serum
H&E	Hematoxylin and eosin
HAS-1	Hyaluronan synthase 1
HAS-2	Hyaluronan synthase 2
HAS-3	Hyaluronan synthase 3
HDF	Human dermal fibroblasts
HEK	Human epidermal keratinocytes
IL-1β	Interleukin-1 beta
IL-6	Interleukin-6
IL-8	Interleukin-8
IFN-γ	Interferon-gamma
JNK	c-Jun N-terminal kinase
MAPKs	Mitogen-activated protein kinases
MMP-1	Matrix metalloproteinase-1
MMP-2	Matrix metalloproteinase-2
MMP-9	Matrix metalloproteinase-9
MMPs	Matrix metalloproteinases
NF-κB	Nuclear factor kappa B
NO	Nitric oxide
p38	p38 mitogen-activated protein kinase
p65	NF-κB p65 subunit (RelA)
p-c-Jun	Phosphorylated c-Jun
p-ERK	Phosphorylated extracellular signal-regulated kinase
p-JNK	Phosphorylated c-Jun N-terminal kinase
p-p38	Phosphorylated p38 mitogen-activated protein kinase
p-p65	Phosphorylated NF-κB p65
PGE2	Prostaglandin E2
qRT-PCR	Quantitative real-time polymerase chain reaction
ROS	Reactive oxygen species
TNF-α	Tumor Necrosis Factor-alpha
UV	Ultraviolet radiation
