# Supplementary material for: Mandelamide Isolated from Prunus persica Flowers Attenuates TNF-α–Driven Oxidative and Inflammatory Responses in Human Skin Cells
Source: Biomolecules. 2026 May 1;16(5):672. doi: 10.3390/biom16050672 (PMC13204222; doi:10.3390/biom16050672)

Figure S1. ^{1}H-NMR spectrum of compound **1** (500MHz, MeOD-d_4)

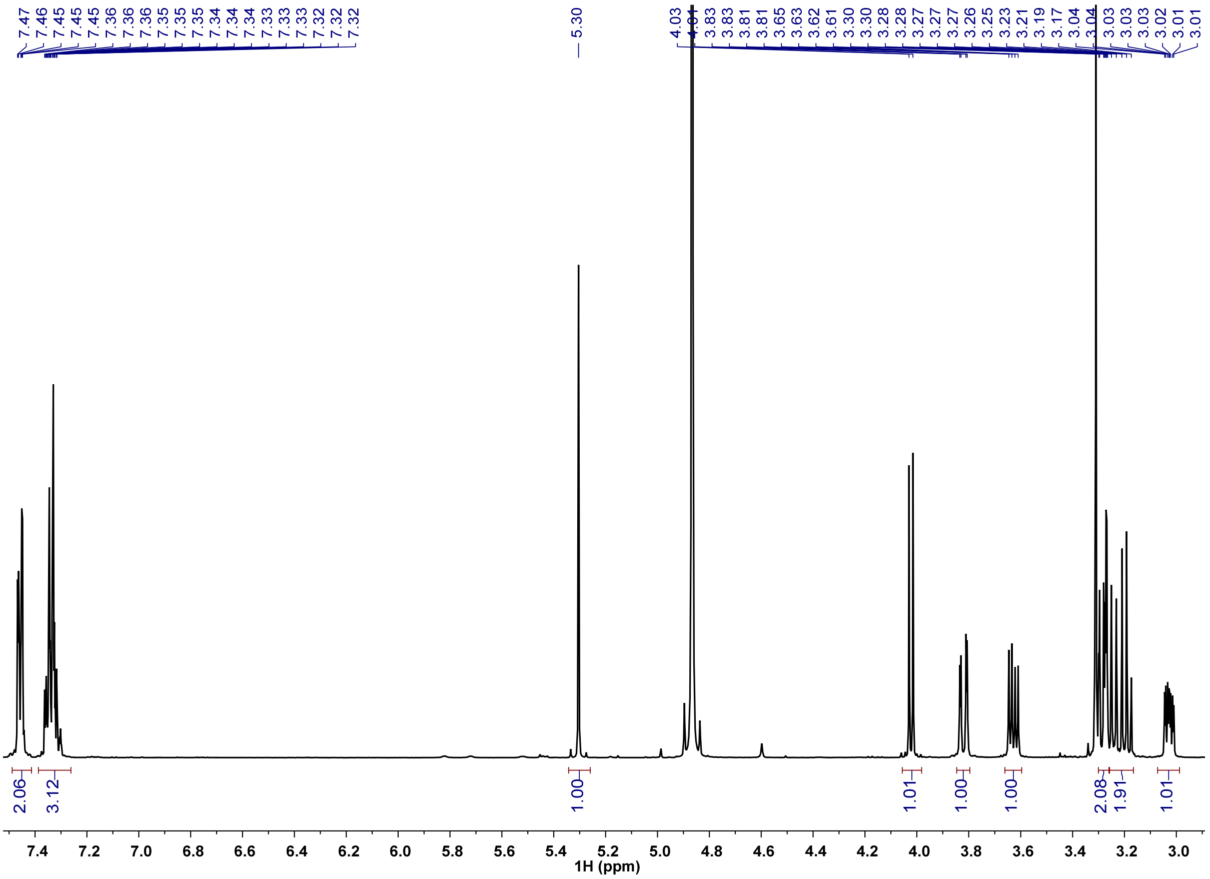

Figure S2. ^{13}C-NMR spectrum of compound **1** (125 MHz, MeOD-d_4)

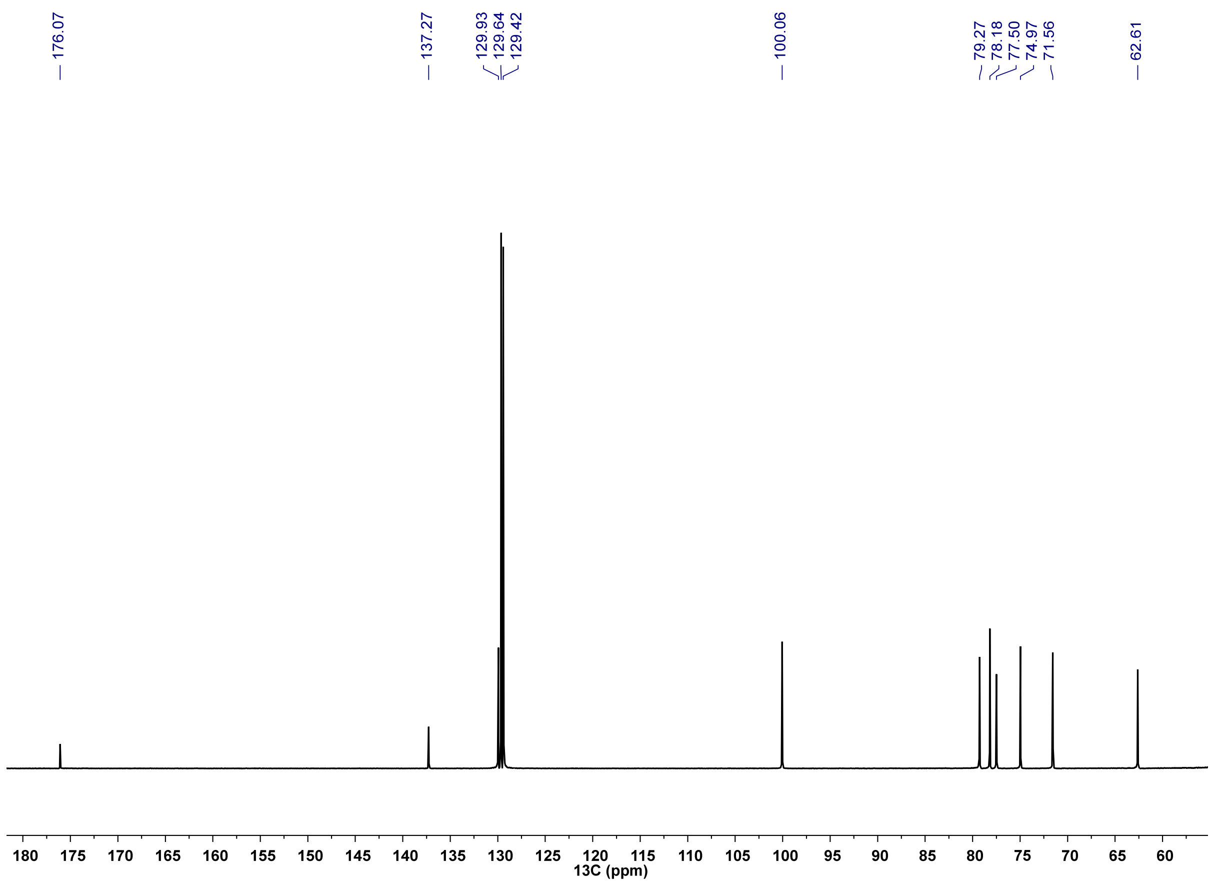

Figure S3. ^{1}H-NMR spectrum of compound **2** (500MHz, D_2O)

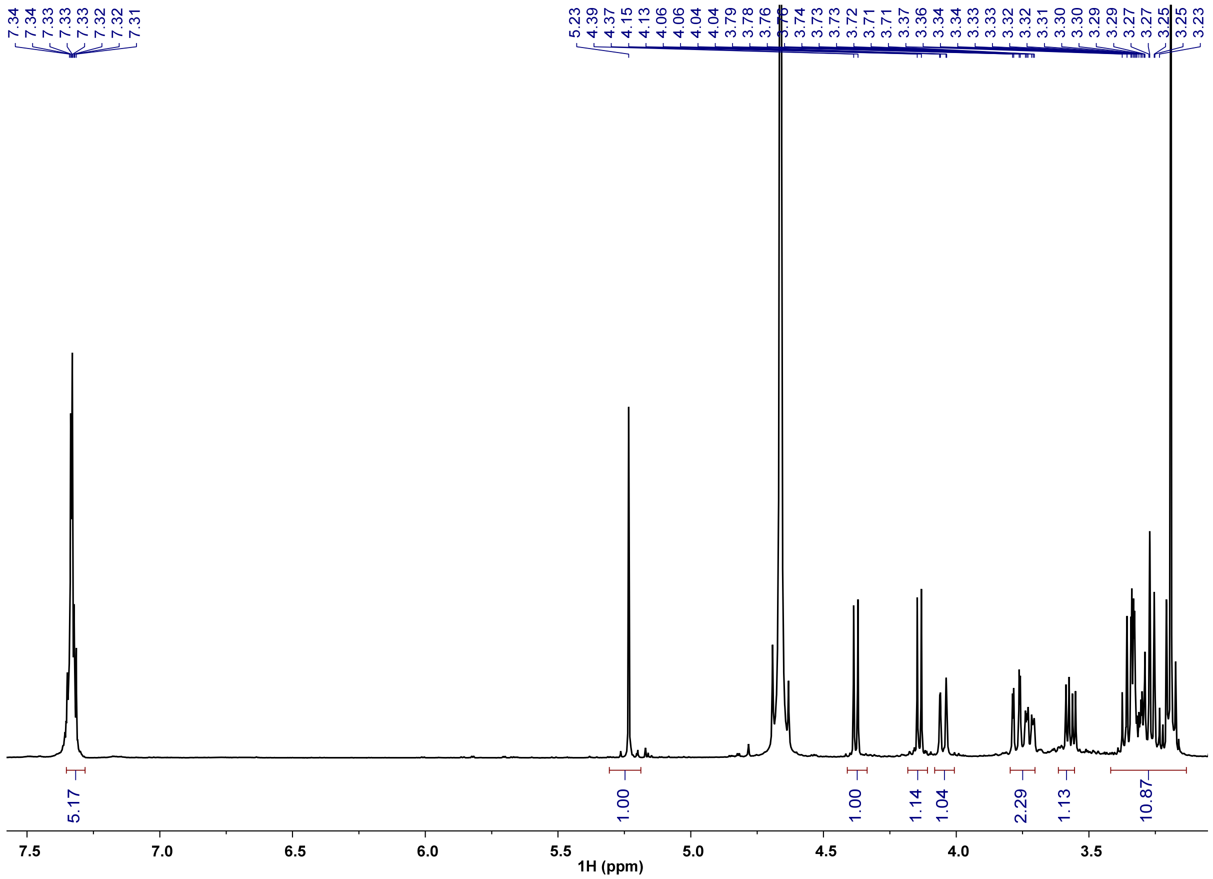

Figure S4. ^{13}C-NMR spectrum of compound **2** (125 MHz, D_2O)

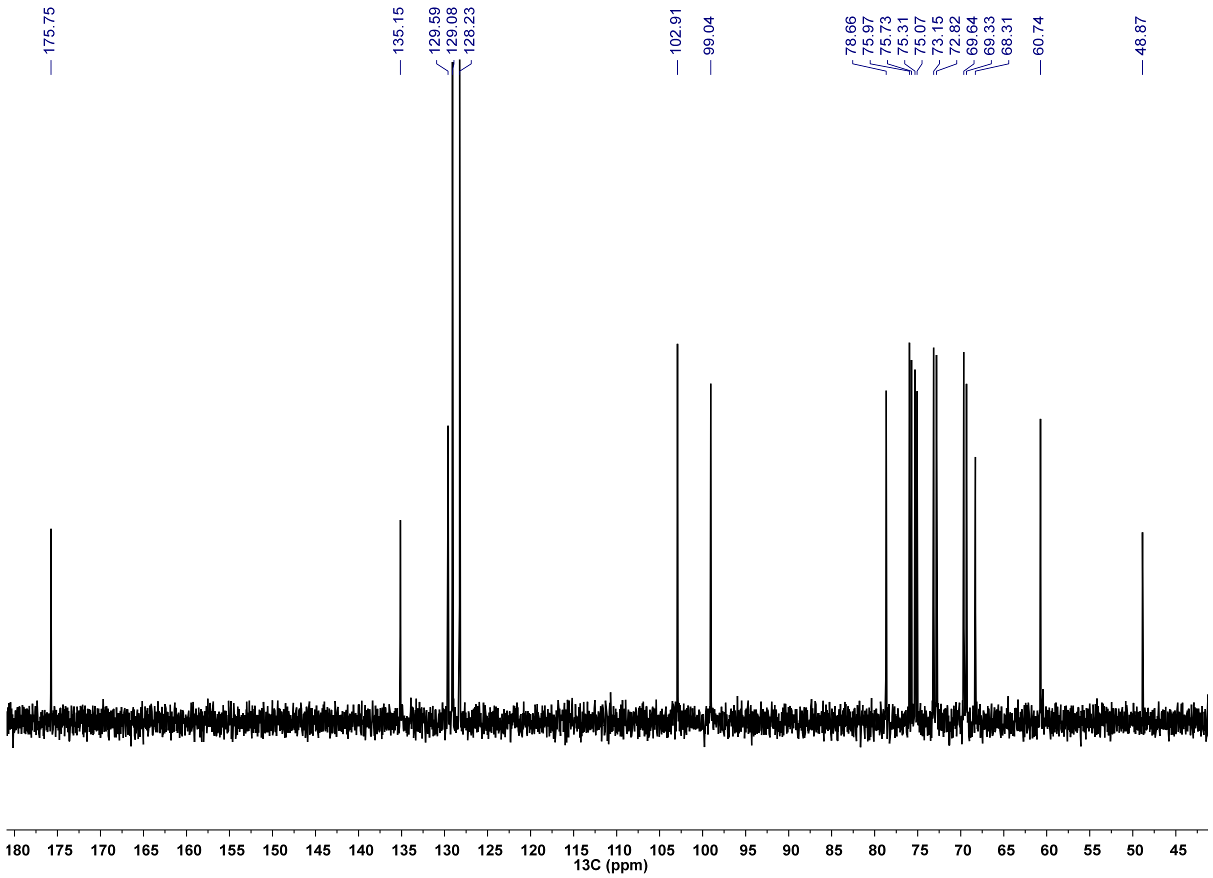

Figure S5. ^{1}H-NMR spectrum of compound **3** (500MHz, MeOD-d_4)

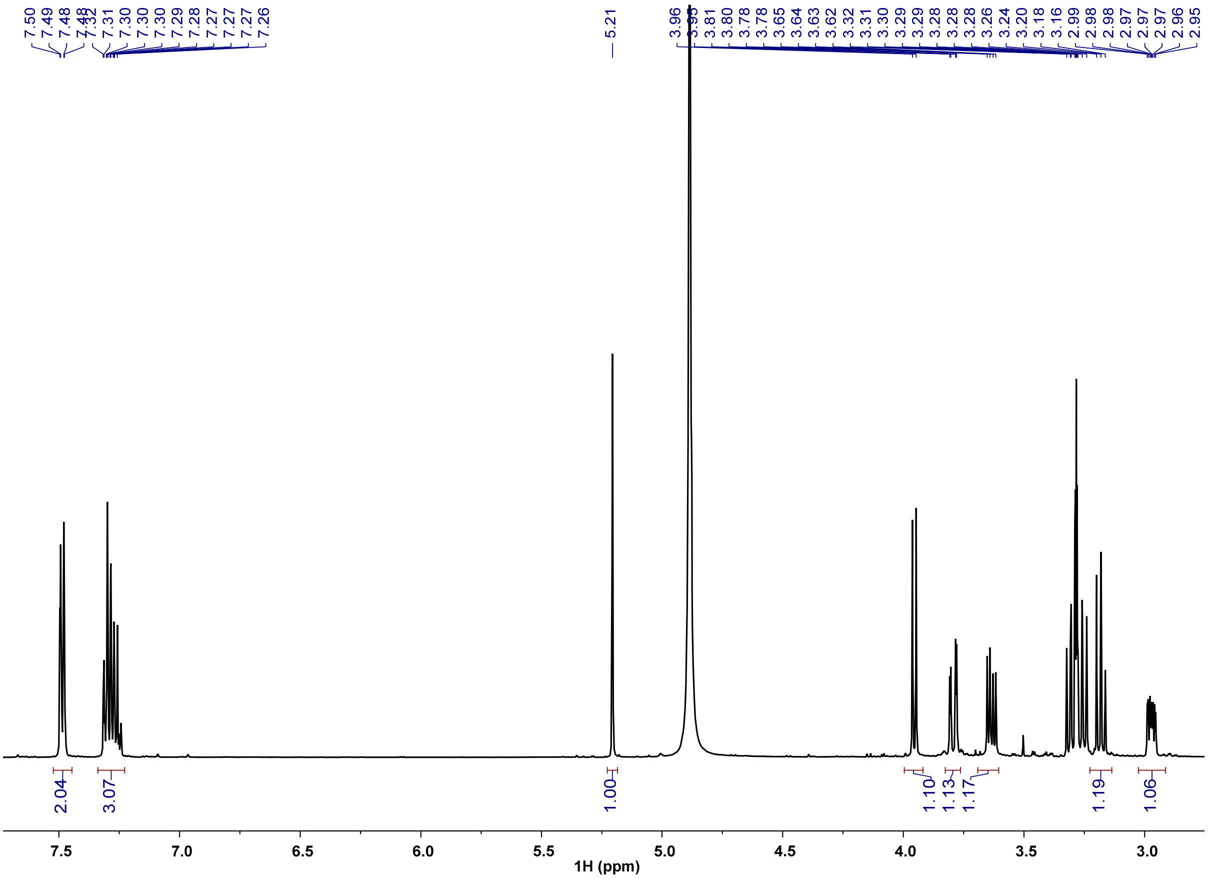

Figure S6. ^{13}C-NMR spectrum of compound **3** (125 MHz, MeOD-d_4)

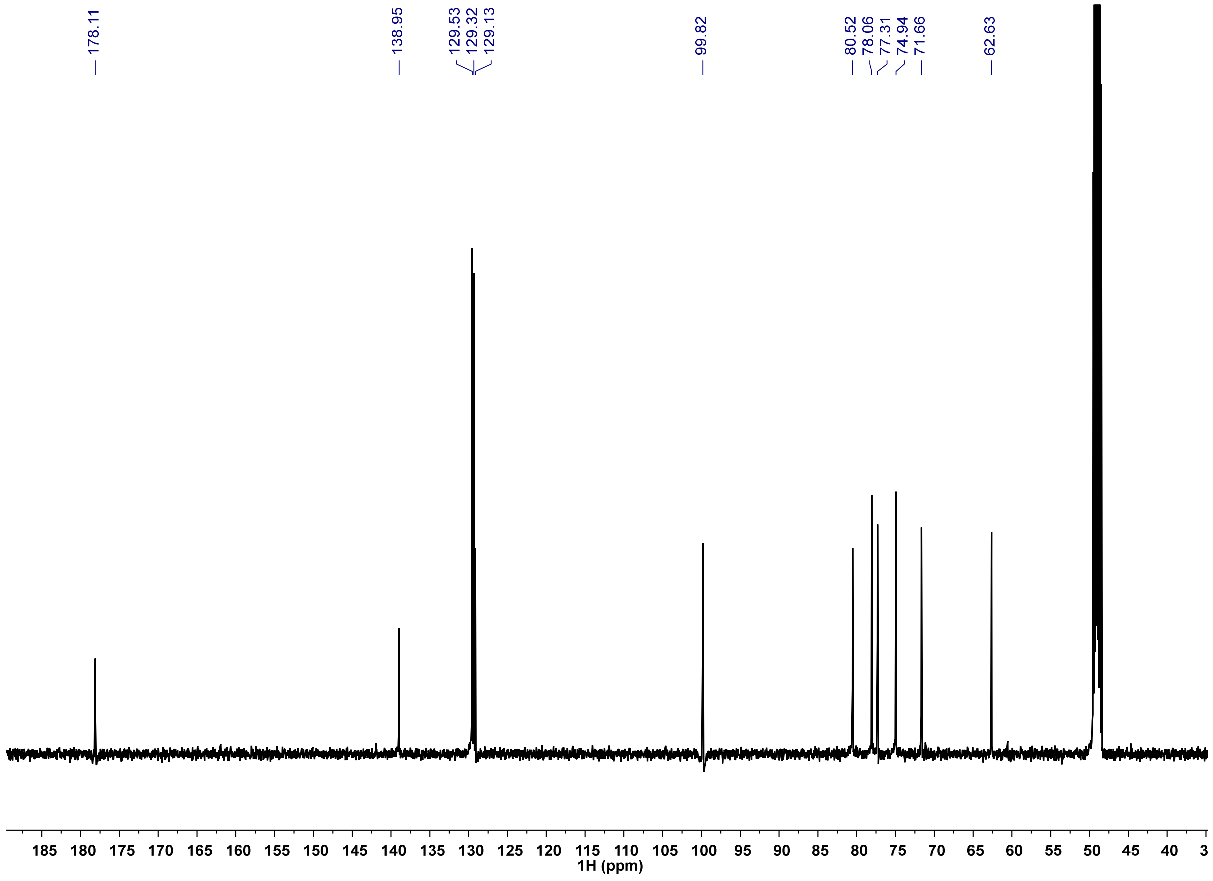

Figure S7. ^{1}H-NMR spectrum of compound **4** (500MHz, D_2O)

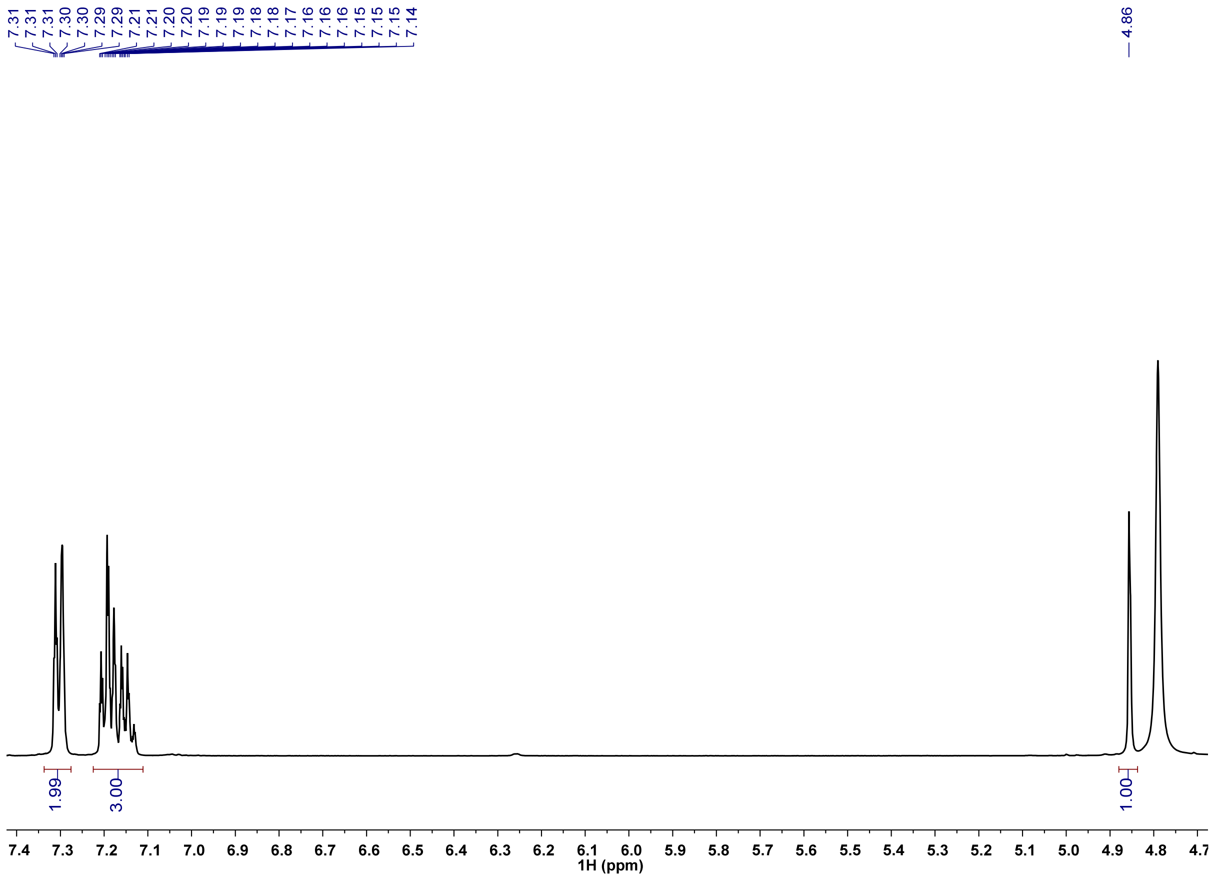

Figure S8. ^{13}C-NMR spectrum of compound **4** (125MHz, D_2O)

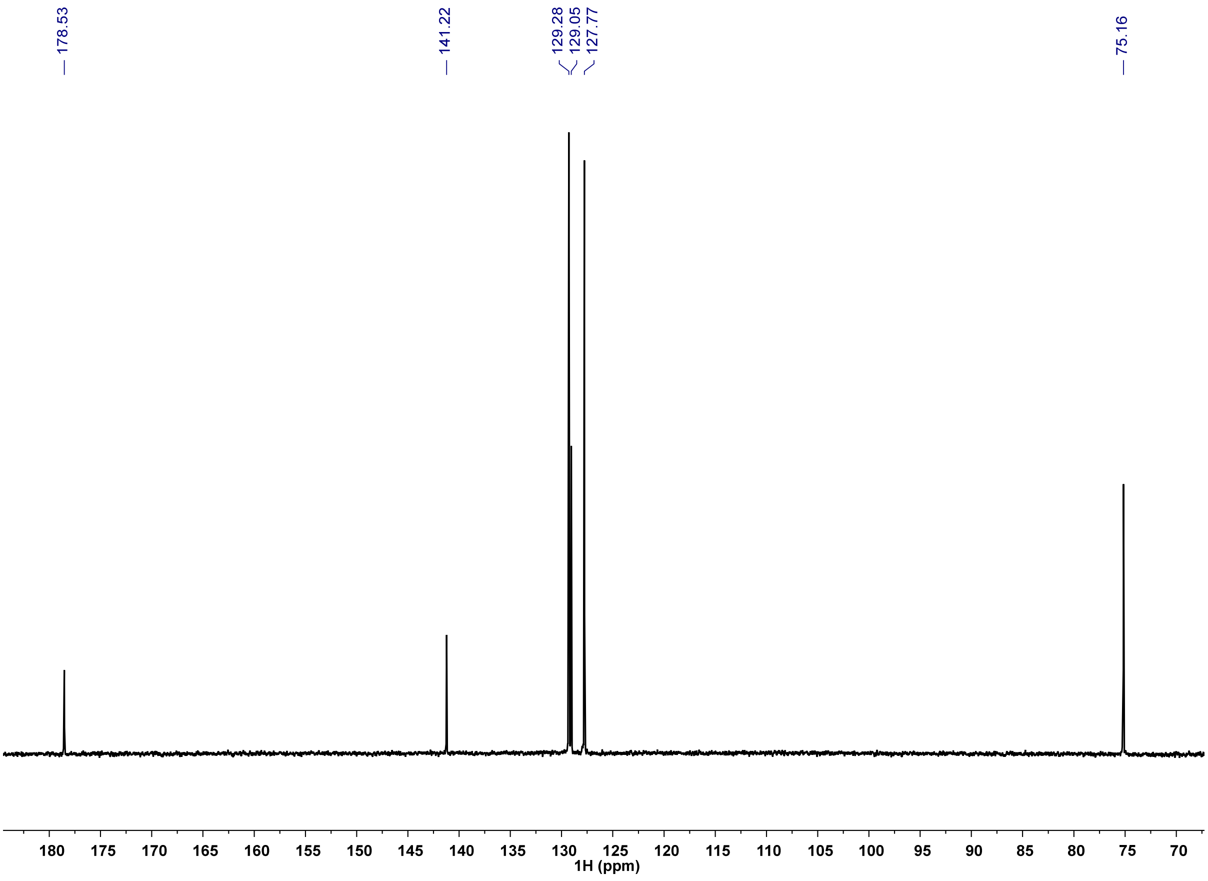

Figure S9. Effects of the *Prunus persica* flower extract and compounds **1**–**4** on cell viability in human dermal fibroblasts (HDFs)

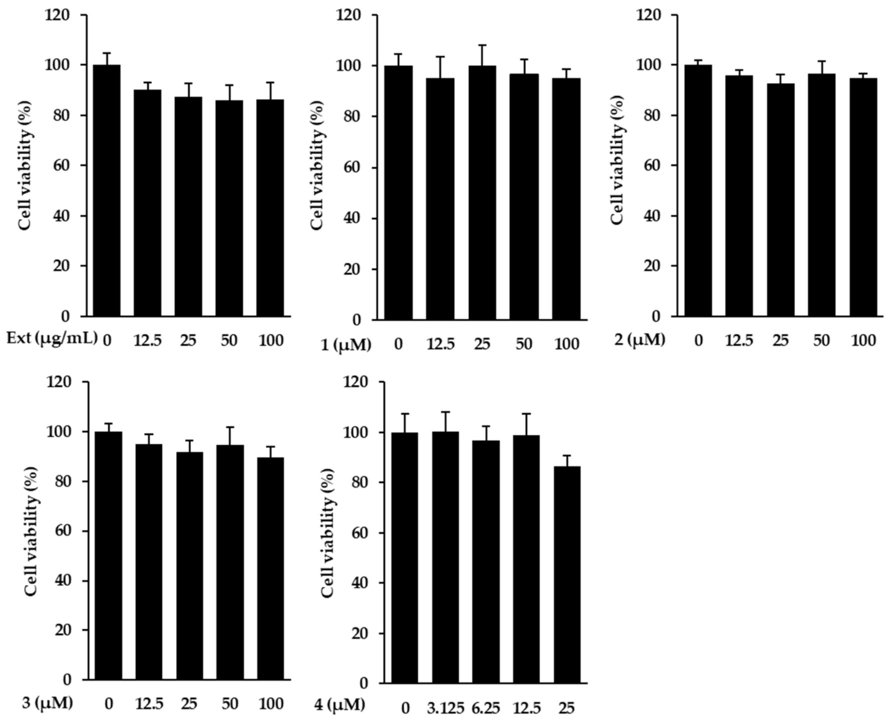

Figure S10. Effects of the *Prunus persica* flower extract and compounds **1**–**4** on MMP-1 secretion in human dermal fibroblasts (HDFs)

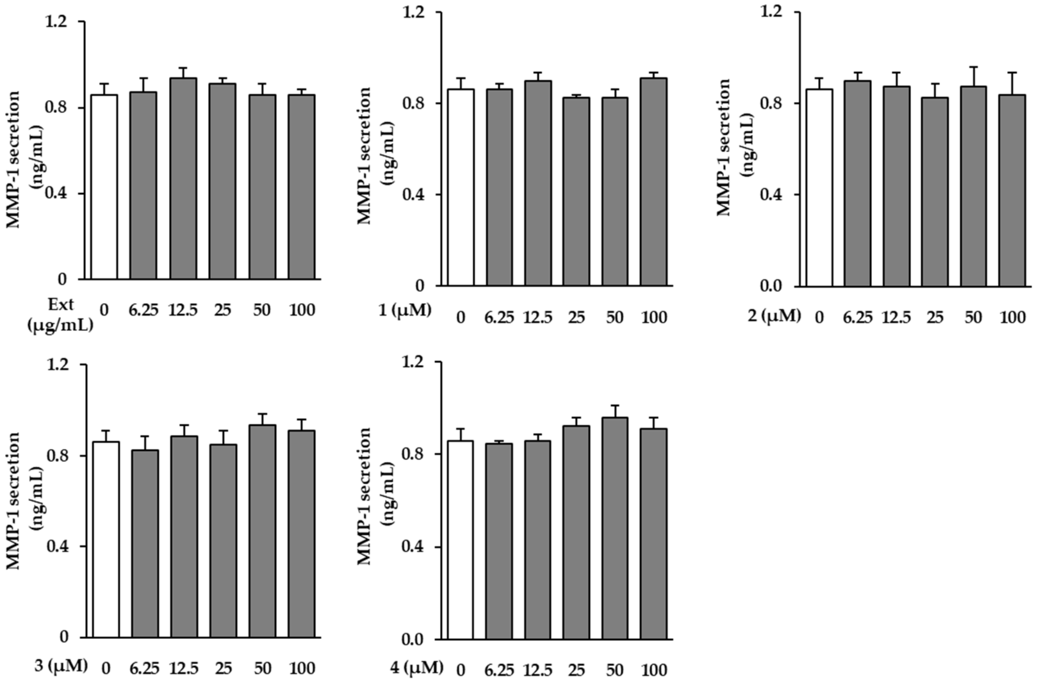

Supplement: Supplementary file 1 [file biomolecules-16-00672-s001.zip › biomolecules-4165713-supplementary figures.pdf]
