# Supplementary figures and images for: Mandelamide Isolated from Prunus persica Flowers Attenuates TNF-α–Driven Oxidative and Inflammatory Responses in Human Skin Cells
Source: Biomolecules. 2026 May 1;16(5):672. doi: 10.3390/biom16050672 (PMC13204222; doi:10.3390/biom16050672)

|               |   |   |      |    |    |     |
|---------------|---|---|------|----|----|-----|
| TNF- $\alpha$ | - | + | +    | +  | +  | +   |
| 4 ( $\mu$ M)  | - | - | 12.5 | 25 | 50 | 100 |

p-JNK

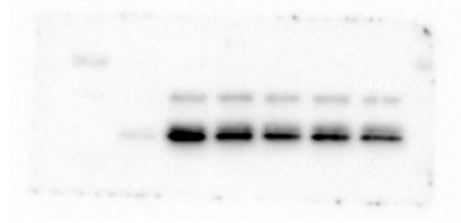

← 54kDa  
← 46kDa

JNK

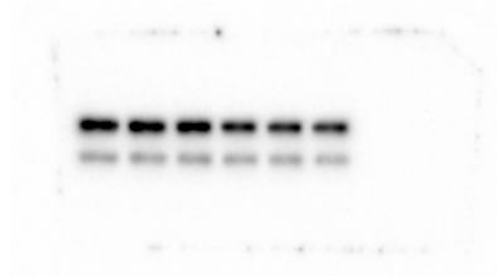

← 54kDa  
← 46kDa

p-ERK

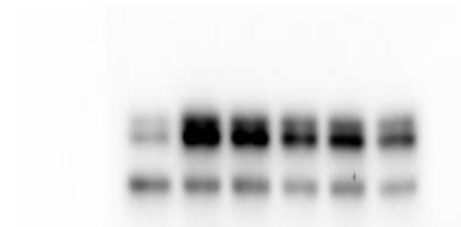

← 44kDa  
← 42kDa

ERK

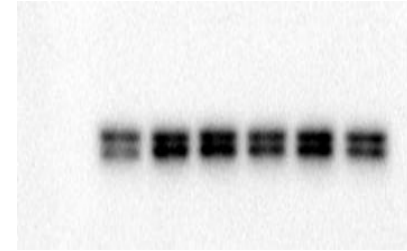

← 44kDa  
← 42kDa

p-p38

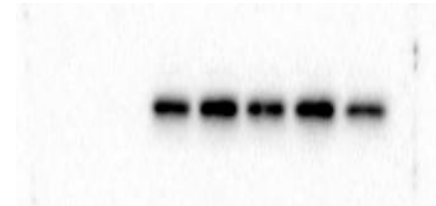

← 43kDa

p38

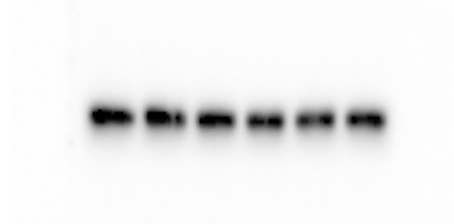

← 43kDa

GAPDH

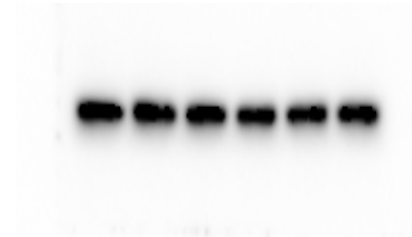

← 37kDa

Supplement: Supplementary file 1 [file biomolecules-16-00672-s001.zip › biomolecules-4165713-file S1.WB.pdf]
